# A Simple, Practical, Surgical Research

**Published:** 1917-11

**Authors:** 


### A Simple, Practical, Surgical Research.

So far as we have been able to learn, there are no accurate statistics on a large scale, showing the actual and relative weights of limbs. In metabolic problems, the question often arises how to allow properly for the obviously different requirements of muscular and skeletal parts, as compared with the viscera. In the case of persons who have suffered amputations, somewhat the same problem arises in a simpler form, to determine relatively normal weight, departures from nutritive requirements, etc. The war furnishes an opportunity to acquire statistics in a short time and we recommend this matter to the consideration of military surgeons. If we had merely the total weight and height of men and the weights of limbs amputated at specified distances, the statistics could readily be assembled.

